# Systematic Performance Comparison of Fe^3+^/Fe^0^/Peroxymonosulfate and Fe^3+^/Fe^0^/Peroxydisulfate Systems for Organics Removal

**DOI:** 10.3390/ma14185284

**Published:** 2021-09-14

**Authors:** Wen-Da Oh, Yeek-Chia Ho, Mardawani Mohamad, Chii-Dong Ho, Rajiv Ravi, Jun-Wei Lim

**Affiliations:** 1 School of Chemical Sciences, Universiti Sains Malaysia, Gelugor 11800, Penang, Malaysia; 2 Civil and Environmental Engineering Department, Centre for Urban Resource Sustainability, Institute of Self-Sustainable Building, Universiti Teknologi PETRONAS, Seri Iskandar 32610, Perak Darul Ridzuan, Malaysia; 3 Faculty of Bioengineering and Technology, Universiti Malaysia Kelantan, Jeli Campus, Jeli 17600, Kelantan, Malaysia; mardawani.m@umk.edu.my; 4 Department of Chemical and Materials Engineering, Tamkang University, Tamsui, New Taipei 251, Taiwan; cdho@mail.tku.edu.tw; 5 School of Applied Sciences, Faculty of Integrated Life Science, Quest International University, Ipoh 30250, Perak, Malaysia; rajiv.ravi@qiu.edu.my; 6 Department of Fundamental and Applied Sciences, HICoE-Centre for Biofuel and Biochemical Research, Institute of Self-Sustainable Building, Universiti Teknologi PETRONAS, Seri Iskandar 32610, Perak Darul Ridzuan, Malaysia; junwei.lim@utp.edu.my

**Keywords:** zero-valent iron, acid orange 7, ferric ion, sulfate radicals, peroxymonosulfate, peroxydisulfate

## Abstract

Activated zero-valent iron (Ac-ZVI) coupled with Fe^3+^ was employed to activate peroxymonosulfate (PMS) and peroxydisulfate (PDS) for acid orange 7 (AO7) removal. Fe^3+^ was used to promote Fe^2+^ liberation from Ac-ZVI as an active species for reactive oxygen species (ROS) generation. The factors affecting AO7 degradation, namely, the Ac-ZVI:Fe^3+^ ratio, PMS/PDS dosage, and pH, were compared. In both PMS and PDS systems, the AO7 degradation rate increased gradually with increasing Fe^3+^ concentration at fixed Ac-ZVI loading due to the Fe^3+^-promoted liberation of Fe^2+^ from Ac-ZVI. The AO7 degradation rate increased with increasing PMS/PDS dosage due to the greater amount of ROS generated. The degradation rate in the PDS system decreased while the degradation rate in the PMS system increased with increasing pH due to the difference in the PDS and PMS activation mechanisms. On the basis of the radical scavenging study, sulfate radical was identified as the dominant ROS in both systems. The physicochemical properties of pristine and used Ac-ZVI were characterized, indicating that the used Ac-ZVI had an increased BET specific surface area due to the formation of Fe_2_O_3_ nanoparticles during PMS/PDS activation. Nevertheless, both systems displayed good reusability and stability for at least three cycles, indicating that the systems are promising for pollutant removal.

## 1. Introduction

Redox oxidation technology involving catalytic persulfate activation has gained significant attention as a promising method to remove anthropogenic pollutants from water. To date, this technology has been successfully used to remove various anthropogenic pollutants in water, including pharmaceuticals, personal care products, industrial waste, and dyes [1,2,3,4]. The two most commonly used persulfates are peroxymonosulfate (PMS) and peroxydisulfate (PDS) [5]. Both PMS and PDS consist of a peroxide bond that can be activated by a catalyst through an electron transfer reaction to produce reactive oxygen species (ROS), such as SO_4_^•–^ and ^•^OH. These ROS have high oxidation potential (E° vs. NHE for SO_4_^•–^ and ^•^OH are 2.5–3.2 and 1.8–2.7 V, respectively [6,7,8]) and can be employed to degrade organic pollutants in water.

Zero-valent iron (Fe^0^, ZVI) is regarded as one of the most versatile catalysts in environmental catalysis [9]. It is relatively efficient, cost-effective, and environmentally friendly [10]. ZVI has been widely used for many applications, including as a catalyst for Fenton oxidation [11] and sequestration [12]. Previously, ZVI has been employed to activate both PMS and PDS to generate ROS for pollutant removal. For instance, Hussain et al. [13] reported that the ZVI/PDS system can be used to generate SO_4_^•–^ and ^•^OH for efficient oxidation of arsenic(III) in water, while Gu et al. [14] reported that efficient removal of 1,1,1-trichloroethane can be achieved using a similar system. For the PMS system, Ghanbari et al. [15] reported that the ZVI/PMS system can be used to efficiently decolorize textile wastewater. These results proved that ZVI has a practical application in environmental remediation.

In general, ZVI activates PDS through sequential steps involving (i) corrosion to produce Fe^2+^ species, and (ii) PDS activation by Fe^2+^ to produce SO_4_^•−^ (Equations (1) and (2)) [9,16]. ZVI can activate PMS via two pathways, namely, corrosion followed by PMS activation (similar to PDS), and direct reaction of ZVI and PMS to produce SO_4_^•−^ and ^•^OH (Equations (3)–(6)) [17].

S_2_O_8_^2−^ + Fe^0^ → **Fe^2+^** + 2SO_4_^2−^ (1)

S_2_O_8_^2−^ + **Fe^2+^** → Fe^3+^ + SO_4_^2−^ + SO_4_^•−^ (2)

2HSO_5_^−^ + Fe^0^ → **Fe^2+^** + 2SO_4_^2−^ + H_2_O (3)

HSO_5_^−^ + **Fe^2+^** → Fe^3+^ + SO_4_^•−^ + H^+^ (4)

HSO_5_^−^ + Fe^0^ + H_2_O → SO_4_^2−^ + **Fe^2+^** + ^•^OH + 2H^+^ + 3e^−^ (5)

HSO_5_^–^ + Fe^0^ + H^+^ → SO_4_^•−^ + **Fe^2+^** + H_2_O + e^−^ (6)

In both cases, the Fe^2+^ species appears to serve as the active species for PMS/PDS activation, suggesting that increasing the Fe^2+^ species can improve the performance of the catalytic system. It is hypothesized that the addition of Fe^3+^ species into the catalytic ZVI system can lead to the increase in Fe^2+^ species (Equation (7)) and improve the pollutant degradation [9]:

2Fe^3+^ + Fe^0^ → 3**Fe^2+^** (7)

Fe^3+^ addition can also reduce the unproductive consumption of PDS/PMS for Fe^2+^ generation from ZVI. Currently, an assessment of the impact of Fe^3+^ addition into the catalytic ZVI system is not available. Direct comparisons of the performance of ZVI as a PDS and PMS activator are also relatively limited.

In this study, the performance of ZVI coupled with Fe^3+^ as a PMS and PDS activator for acid orange 7 (AO7) removal was compared. The effects of the ZVI:Fe^3+^ ratio, PDS/PMS dosage, and initial pH on AO7 were systematically evaluated. The main ROS generated from the PMS and PDS systems was also identified. Finally, the extent of mineralization and the reusability of the PMS and PDS systems were evaluated.

## 2. Materials and Methods

### 2.1. Chemicals

The following chemicals were used without further purification in this study: potassium peroxydisulfate (K_2_S_2_O_8_, Merck), Oxone^®^ (source of PMS, 2KHSO_5_, KHSO_4_, K_2_SO_4_, Acros Organics), acid orange 7 (C_16_H_11_N_2_NaO_4_S, Aldrich), nitrobenzene (Qrec), hydrochloric acid (Fisher), ethanol (Qrec), sodium hydroxide (Fisher), iron(III) nitrate nonahydrate (Qrec), and ZVI (Chengdu). Prior to the experiment, the ZVI was activated by exposing the ZVI to 1 M HCl for 10 min (Ac-ZVI).

### 2.2. Performance Study

All the performance studies were conducted using a 250 mL reaction vessel in batches. Typically, a 100 mL solution consisting of 8.5 mg L^−1^ of AO7 and PMS/PDS (at selected concentrations) was prepared and agitated rapidly using a magnetic stirrer. Then, a known amount of Fe^3+^ and Ac-ZVI was added into the reactor to start the reaction. At the selected time interval, sampling was conducted by collecting 3 mL of the solution in the reactor. The AO7 concentration in the collected sample was analyzed using a UV–Vis spectrophotometer (Hitachi U-2000) at λ_max_ = 485 nm. Control studies consisting of only AO7 + PMS, AO7 + PMS + Fe^3+^ or Ac-ZVI, and AO7 only were also conducted. The effects of different Fe^3+^ concentration (0–20 mg L^−1^), Ac-ZVI dosage (1–3 g L^−1^), pH (3–9), and PDS/PMS:AO7 mol ratio (10:1–100:1) on AO7 degradation were studied. The pH was adjusted using diluted HCl and NaOH. The dominant reactive oxygen species (ROS) was identified using chemical scavengers, namely, ethanol (3 M) and nitrobenzene (70 mg L^−1^). During the performance study at the selected condition, ethanol or nitrobenzene was added to inhibit SO_4_^•−^ and ^•^OH, and ^•^OH, respectively. Similarly, at the specific condition, the total organic carbon (TOC), Fe concentration, and extent of reusability of the catalytic system were also determined. Meanwhile, the PMS/PDS concentration was determined using the iodometric method as described previously [18]. The TOC was determined using a TOC analyzer (TOC-L, Shimadzu), while the Fe concentration was quantified using an Atomic Absorption Spectrometer (PerkinElmer AAnalyst 400). The extent of reusability was evaluated by conducting the performance study using the same Ac-ZVI over several cycles. The experiments were conducted in duplicate.

### 2.3. Characterization Study

The characteristics of Ac-ZVI and the used Ac-ZVI in both PMS and PDS systems were investigated using various advanced instruments. The crystal structure of the catalysts was determined using the X-ray diffraction (XRD, Bruker AXS D8 Advance with Cu-Kα source of λ = 1.5418 Å and scan rate of 0.02° s^−1^) method, while the surface functional groups were determined using a Fourier transform infrared (FTIR, Frontier, PerkinElmer, Neuchatel, Switzerland) spectrometer. The Brunauer–Emmett–Teller (BET) specific surface area of the catalysts was determined from the nitrogen adsorption-desorption isotherm obtained using a porosimeter (ASAP 2020, NSW 2229, Australia). The morphology of the Ac-ZVI and the used Ac-ZVI was studied using a scanning electron microscope (SEM, Quanta FEG 650, London, UK).

## 3. Results and Discussion

### 3.1. Performance Evaluation

#### 3.1.1. Effect of Fe^3+^ Concentration

**Figure 1:**
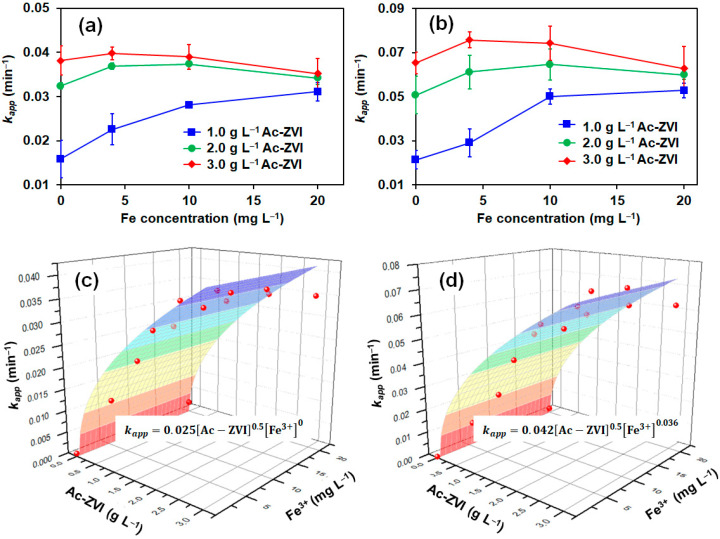
Effect of the Fe^3+^:Ac-ZVI ratio on the AO7 removal rate for (**a**) PMS and (**b**) PDS systems, and the relationship of *k_app_*, Ac-ZVI dosage and Fe^3+^ in the (**c**) PMS and (**d**) PDS systems. Conditions: [AO7] = 8.5 mg L^−1^ and pH = 3–4.

Several control studies involving AO7 removal (a) in the presence of Fe^3+^ only, (b) without a catalyst, and (c) without PMS/PDS) were conducted, indicating that Fe^3+^ alone cannot activate PMS/PDS effectively (<10% AO7 removed in 60 min). Direct PMS/PDS oxidation and adsorption by Ac-ZVI had no significant impact on AO7 removal (<9% AO7 removed in 60 min). The AO7 degradation in the PMS and PDS systems at various time intervals was monitored by varying the Fe^3+^ concentration at fixed Ac-ZVI loading, and the results were fitted into the pseudo first-order kinetics: ${\left[\mathrm{AO}7\right]}_{t}={\left[\mathrm{AO}7\right]}_{o}e^{-k_{app}t}$, where [AO7]_t_ and [AO7]_o_ are the AO7 concentration at various time intervals and the initial AO7 concentration, respectively; and *k_app_* is the pseudo first-order rate constant. The relationship between the calculated *k_app_* value and the Fe^3+^ concentration at various Ac-ZVI loadings in the PMS and PDS systems are shown in Figure 1a,b, respectively. In general, the PMS and PDS systems followed a similar trend where, without Fe^3+^ addition, the *k_app_* value increased gradually with increasing Ac-ZVI loading due to greater catalytic active sites available for PMS/PDS activation [19]. At 1.0 g L^−1^ Ac-ZVI, the *k_app_* value increased with increasing Fe^3+^ concentration from 0 to 20 mg L^−1^ for both PMS and PDS systems. The addition of Fe^3+^ promoted the liberation of Fe^2+^ from the Ac-ZVI through Equation (7), which is beneficial for PMS/PDS activation [9]. However, the quantum of increase diminished with increasing Fe^3+^ for both PMS and PDS systems, and this can be attributed to two factors. First, the higher Fe^3+^ concentration leads to a rapid increase in Fe^2+^ where excessive Fe^2+^ can also act undesirably as an ROS scavenger, reducing the productive consumption of ROS for AO7 removal (Equations (8) and (9)).

Fe^2+^ + SO_4_^•−^ → Fe^3+^ + SO_4_^2−^ (8)

Fe^2+^ + ^•^OH → Fe^3+^ + OH^−^ (9)

Second, excessive Fe^3+^ can compete with other Fe species for reaction with PS and PMS (Equations (10) and (11)), leading to the depletion of available oxidant for SO_4_^•−^ generation.

Fe^3+^ + HSO_5_^−^ → Fe^2+^ + SO_5_^•−^ (10)

Fe^3+^ + S_2_O_8_^2−^ → Fe^2+^ + S_2_O_8_^•−^ (11)

Not surprisingly, these effects were more pronounced at higher Ac-ZVI loading, particularly at 3.0 g L^−1^ where a 4–9% decrease in *k_app_* value was observed in the presence of 20 mg L^−1^ Fe^3+^ compared to that without Fe^3+^ addition attributed to the uncontrolled liberation of Fe^2+^. The results indicate that an optimum Fe^3+^ concentration must be maintained to effectively improve the catalytic performance in both systems.

**Figure 2:**
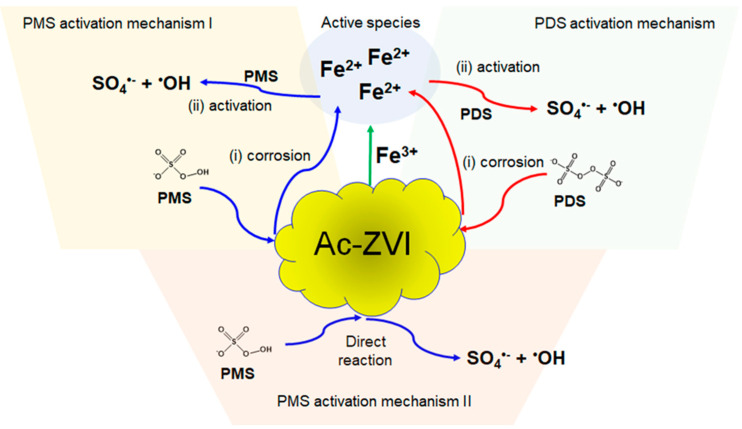
Schematic illustration of the PMS and PDS activation by ZVI.

To compare the effect of Fe^3+^ on the PMS and PDS systems, the *k_app_* value was further expanded to include the specific rate constant (*k_sp_*) as a function of [Fe^3+^] and [Ac-ZVI] (Equation (12)).

(12) $$-\frac{d\left[\mathrm{AO}7\right]}{\mathrm{dt}}=k_{app}\left[\mathrm{AO}7\right]=k_{sp}{\left[\mathrm{Ac}-\mathrm{ZVI}\right]}^{0.5}{\left[{\mathrm{Fe}}^{3+}\right]}^{n}\left[\mathrm{AO}7\right]$$

where n is the order of reaction for Fe^3+^. The reaction order of [Ac-ZVI] was set at 0.5 based on the preliminary fitting where the reaction order of 0.5 provided a reasonably good fit. To avoid error due to too many parameters, the reaction order of [Ac-ZVI] was fixed at 0.5. The kinetic fittings (Figure 1c,d) revealed that the n values are 0 and 0.036 for the PMS and PDS systems, respectively, while their corresponding *k_sp_* values are 0.024 and 0.042, respectively. The relatively lower n value in the PMS system indicates that the Fe^3+^ did not significantly improve the PMS system compared to the PDS system. Comparison of the *k_sp_* values showed that the Ac-ZVI/Fe^3+^ system is more efficient in activating PDS than PMS. The trends in the n and *k_sp_* values are reasonable based on the possible activation pathways of PDS and PMS. In the PDS system, only one pathway exists, where the ROS generation must be preceded by a rate-limiting step involving Ac-ZVI corrosion and Fe^2+^ dissolution from the Ac-ZVI surface [20]. In the PMS system, the ROS generation can occur via two pathways, namely, through a similar pathway and directly from the reaction between PMS and Ac-ZVI. Consequently, Fe^2+^, as an active species, is more important for the PDS system than the PMS system. The schematic illustration of the reaction pathway is presented in Figure 2. However, despite more reaction pathways being available in the PMS system, PDS activation is superior due to (i) the higher redox potential of PDS (E° = 2.01 V vs. NHE) compared to PMS (E° = 1.92 V vs. NHE), and (ii) the single pathway allows more controlled Fe^2+^ liberation, promoting productive Fe^2+^ consumption.

#### 3.1.2. Effect of PMS/PDS Dosage

**Figure 3:**
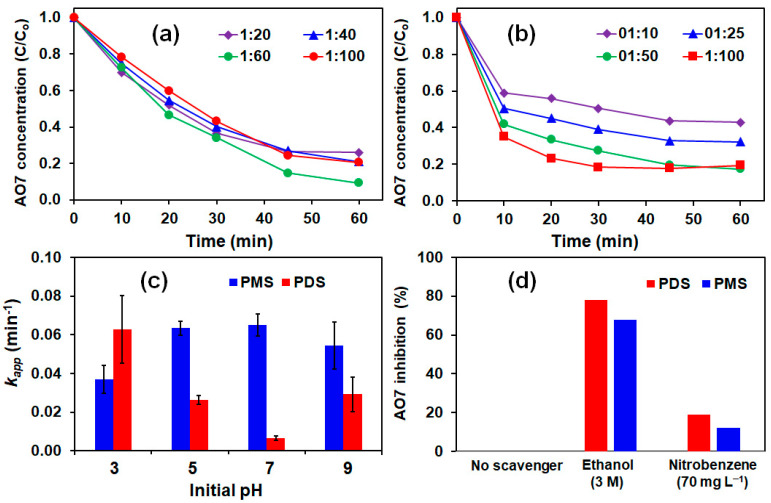
Effects of (**a**) PMS dosage; (**b**) PDS dosage; (**c**) pH; and (**d**) radical scavengers on AO7 removal. Conditions: [Ac-ZVI] = 2 g L^−1^, [Fe^3+^] = 4 mg L^−1^ and [PMS]/[PDS] = (for (**c**,**d**)).

The effect of PMS and PDS dosage was investigated at the optimum Fe^3+^:Ac-ZVI ratio. Figure 3a,b shows the effect of PMS/PDS dosage on AO7 degradation. Apparently, increasing the AO7:PMS ratio from 1:20 to 1:60 did not result in a significant change in the AO7 degradation rate (*k_app_* = 0.030–0.037 min^−1^). Further increase in the AO7:PMS ratio from 1:60 to 1:100 resulted in a minor decrease in *k_app_* to 0.025 min^−1^ due to the increased probability of the scavenging effect and competition reactions involving PMS and generated ROS to produce weaker ROS (Equations (13)–(17)) [21].

HSO_5_^−^ + SO_4_^•−^ → SO_5_^•−^ + SO_4_^2−^ + H^+^ (13)

HSO_5_^−^ + OH^•^ → SO_5_^•−^ + H_2_O (14)

SO_4_^•^^−^ + SO_4_^•^^−^ → S_2_O_8_^2^^−^ (15)

OH^•^ + OH^•^ → H_2_O_2_ (16)

SO_4_^•^^−^ + OH^•^ → HSO_5_^−^ (17)

In the PDS system, increasing the AO7:PDS ratio from 1:10 to 1:100 led to a gradual increase in the *k_app_* value from 0.034 to 0.079 min^−1^. At a higher AO7:PDS ratio, the greater PDS concentration can interact more efficiently with Ac-ZVI to generate more Fe^2+^ for PDS activation and, subsequently, ROS generation [22]. Comparison of the PMS and PDS systems showed that the oxidant dosage had a greater influence in the PDS system, and again, this is attributed to the difference in their activation mechanisms. Because the PDS activation involves only one pathway, the ROS production is more controlled compared to the PMS system and is limited by the PDS dosage. In the PMS system, the reactions involved are more direct and ROS production is limited by the available Ac-ZVI surface area. Direct ROS generation may produce excessive ROS, resulting in an increase in the scavenging effect and competition reactions (Equations (13)–(17)).

#### 3.1.3. Effect of pH

The pH of the solution plays a critical role in influencing the performance of the system. As indicated in Figure 3c, the *k_app_* value in the PDS system decreased from 0.063 to 0.007 min^−1^ with increasing pH from 3 to 7. At acidic pH, the ZVI corrosion rate is higher, leading to increased Fe^2+^ liberation and ROS generation [23]. As pH increases to 7, the ZVI corrosion rate decreases (less Fe^2+^ liberation), inhibiting the ROS generation rate. The pH change after the reaction for all pHs was within the ±2 limit. The generated Fe^2+^ was also precipitated at pH > 6 as Fe(OH)_2_, further restricting Fe^2+^ from participating in the PDS activation reaction [24]. However, as the initial pH was increased from 7 to 9, the *k_app_* value increased to 0.02 min^−1^, which could be due to the base activation of PDS [25,26]. Unlike the PDS system, the *k_app_* value for the PMS system increased from 0.037 at pH 3 to 0.055–0.065 min^−1^ at pH > 5. As indicated above, the PMS activation depends on the Ac-ZVI surface area, and its degradation is less affected by the Ac-ZVI corrosion rate and Fe^2+^ precipitation. Hence, a reduced corrosion rate will not significantly influence the performance of the PMS system. This suggests that the PMS system is less influenced by the pH change.

### 3.2. Identification of Dominant Radical

The dominant radical was identified using radical scavengers, namely ethanol and nitrobenzene, to scavenge the selected ROS. The concentration of chemical scavengers used was selected based on their reactivity (estimated from the second-order rate constant) with their respective SO_4_^•−^ and ^•^OH. Ethanol can be used to scavenge both SO_4_^•−^ (second-order rate constant, $k_{SO_{4}^{•–}+Ethanol}$ = 1.6–7.7 × 10^7^ M^−1^ s^−1^ ) and ^•^OH ($k_{HO^{•}+Ethanol}$ = 1.2–2.8 × 10^9^ M^−1^ s^−1^), while nitrobenzene can scavenge ^•^OH ($k_{HO^{•}+nitrobenzene}$ = 3.9 × 10^9^ M^−1^ s^−1^) but not SO_4_^•−^ ($k_{SO_{4}^{•–}+nitrobenzene}$ ≤ 10^6^ M^−1^ s^−1^) [27]. At 3 M ethanol, it is sufficient to scavenge all the generated radicals, while at 70 mg L^−1^ nitrobenzene, it is sufficient to scavenge all the ^•^OH without affecting AO7 degradation due to SO_4_^•−^. Figure 3d shows the percentage of inhibition of AO7 degradation (calculated based on the *k_app_*) by ethanol and nitrobenzene in the PDS and PMS systems. In both systems, 70–80% of the degradation rate was inhibited by ethanol, while only <20% of the degradation rate was inhibited by nitrobenzene, indicating that both SO_4_^•−^ and ^•^OH contributed to the degradation, with SO_4_^•−^ playing the dominant species. It should be noted that the results are strictly pH-dependent since the distribution of ROS can change with pH. For instance, SO_4_^•−^ can be converted to ^•^OH in the presence of H_2_O and HO^−^ [9,28].

### 3.3. Recyclability, Extent of Mineralization, and Post Mortem Catalyst Analysis

**Figure 4:**
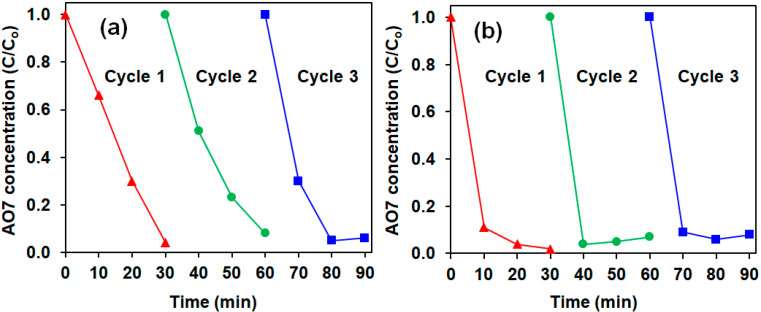
Reusability of Ac-ZVI for (**a**) PMS and (**b**) PDS systems. Conditions: [AO7] = 8.5 mg L^−1^, [ZVI] = 2 g L^−1^, [Fe^3+^] = 4 mg L^−1^, [PMS/PDS] = 0.5 g L^−1^, pH = 3–4.

The reusability of the Ac-ZVI/Fe^3+^ in both PMS and PDS systems is presented in Figure 4, indicating that the Ac-ZVI/Fe^3+^ system can be reused for at least three cycles without significant loss in its catalytic efficiency (>95% AO7 removed in 30 min). It was also observed that the Fe leaching was only 0.35–0.36% from the Ac-ZVI, indicating that the system is stable and can be used for continuous AO7 degradation. The TOC removal efficiency for PMS and PDS systems was 51% and 43%, respectively, and the PMS/PDS consumption was 54% ± 6% after 4 h, indicating that mineralization of the AO7 molecules into innocuous compounds, such as CO_2_ and H_2_O, can occur efficiently. Since the residual PMS/PDS was still present after 4 h, higher mineralization efficiency can still be achieved by increasing the reaction time. A brief comparison (Table 1) of the results obtained in this study using the Ac-ZVI/Fe^3+^ system with other catalytic systems for AO7 removal revealed that the Ac-ZVI/Fe^3+^ system can provide a comparable or better treatment efficiency. This indicates that the Ac-ZVI/Fe^3+^ system has promising potential as a PMS/PDS activator for environmental applications.

**Figure 5:**
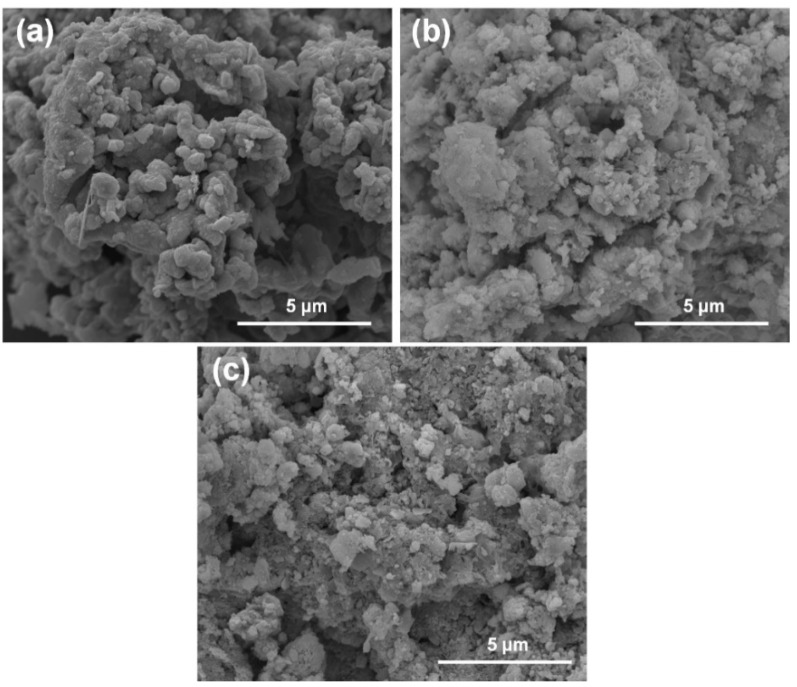
FESEM micrographs of (**a**) Ac-ZVI; (**b**) Ac-ZVI after PMS oxidation; (**c**) Ac-ZVI after PDS oxidation.

**Figure 6:**
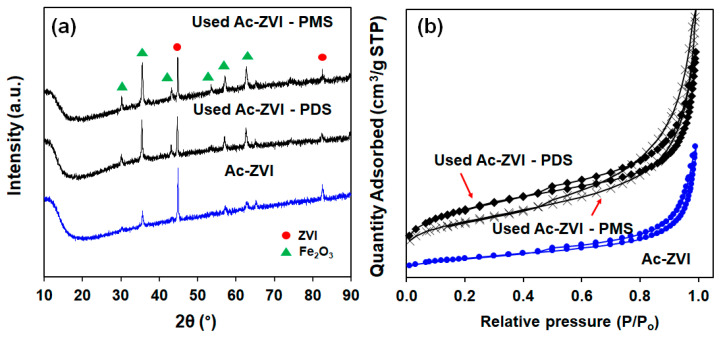

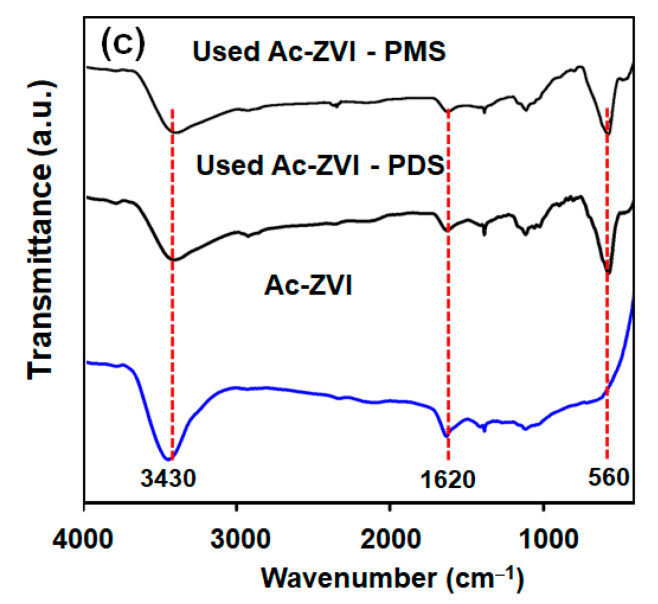
(**a**) XRD patterns; (**b**) nitrogen adsorption-desorption isotherms; and (**c**) FTIR spectra of pristine and used Ac-ZVI.

The FESEM micrographs of pristine and used Ac-ZVI in Figure 5 indicate that after PMS/PDS activation, the smooth Ac-ZVI surface became coarser and various nanoparticles attached on the Ac-ZVI surface could be observed. Not surprisingly, this is due to the PDS/PMS interaction with Ac-ZVI, forming Fe_2_O_3_. The XRD patterns (Figure 6a) of the pristine and used Ac-ZVI confirmed the formation of Fe_2_O_3._ There is no discernible difference between the XRD patterns of the used Ac-ZVI in both PMS and PDS systems. The N_2_ adsorption-desorption isotherms (Figure 6b) revealed that the pristine and used Ac-ZVI is a mesoporous material that consists of a type-IV isotherm with an H3 hysteresis loop. This indicates that the catalyst consists of a wide range of pore sizes. As a result of Fe_2_O_3_ formation, the BET specific surface area increased from 8.7 to >30 m^2^ g^−1^. The FTIR spectrum of pristine and used Ac-ZVI in Figure 6c shows the characteristic vibration bands at 1620 and 3430 cm^−1^ which can be ascribed to the FeOOH stretching view and surface OH group, respectively [34,35]. After use, an additional peak at 560 cm^−1^ was observed due to the Fe-O bond of Fe_2_O_3_ [18]. The post mortem catalyst characterization showed that, regardless of the changes in the characteristics of the Ac-ZVI after use in both PMS and PDS systems, the performance of the systems remains unaffected.

## 4. Conclusions

In summary, Ac-ZVI coupled with Fe^3+^ was successfully used to activate PMS and PDS for AO7 removal. The effects of the Ac-ZVI:Fe^3+^ ratio, PMS/PDS dosage, and pH were compared. The results showed that, in general, increasing the Ac-ZVI:Fe^3+^ ratio produced a promotional effect on the performance of the catalytic system. However, when Fe^3+^ was in excess, the performance deteriorated due to the self-scavenging effect and competitive reactions. Increasing the PMS/PDS dosage generally leads to improved performance, while varying the pH leads to poorer performance in the PDS system but better performance in the PMS system due to the difference in their degradation mechanisms. The PMS system is less influenced by pH changes. The dominant ROS in both systems was identified to be SO_4_^•−^. Overall, the Ac-ZVI coupled with Fe^3+^ can be used for at least three cycles without significant loss in catalytic activity, indicating that it is promising for environmental remediation.

## Figures and Tables

**Table 1:** A comparison of Ac-ZVI/Fe^3+^ system with other catalysts for AO7 removal.

Catalyst	Oxidant	Performance	Ref.
CuO	PMS	>85% of 20 mg L^−1^ AO7 removed in 60 min, 0.05 g L^−1^ CuO and 0.1 mM PMS	[29]
FeOOH	PMS	>95% of 50 mg L^−1^ AO7 removed in 30 min, 0.3 g L^−1^ FeOOH and 20:1 PMS/AO7 molar ratio	[30]
MnCeO_X_	PDS	>80% of 10 mg L^−1^ AO7 removed in 120 min, pH = 6.8, 0.7 g L^−1^ MnCeO_X_ and 4 mmol L^−1^ PDS.	[31]
Activated carbon	PMS	100% of 100 mg L^−1^ AO7 removed in 60 min, 0.30 g L^−1^ GAC and 20:1 PMS/AO7 molar ratio	[32]
nano-Co_3_O_4_	PMS	>98% of 0.2 mM AO7 removed in 30 min, 0.5 g L^−1^ nano-Co_3_O_4_ and 2 mM PMS	[33]
Ac-ZVI/Fe^3+^	PMS/PDS	>98% of 8.5 mg L^−1^ AO7 removed in 30 min with [ZVI] = 2 g L^−1^, [Fe^3+^] = 4 mg L^−1^, [PMS/PDS] = 0.5 g L^−1^, pH = 3–4	This study

## Data Availability

Will be provided upon request.
